# The statue of Matron Alice Cashin (1870–1939)

**DOI:** 10.1177/09677720241273695

**Published:** 2024-09-02

**Authors:** Hareesha Rishab Bharadwaj, Yukti Karki

**Affiliations:** 1 Faculty of Biology Medicine and Health, 5292 The University of Manchester, Manchester, UK; 2 128550 Faculty of Medicine, Kathmandu Medical College, Kathmandu, Nepal

**Keywords:** Matron, Australia, UK, healthcare, tribute

## Abstract

Alice Alanna Cashin (1870–1939) was a pioneering Australian nurse whose career spanned both conflict and humanitarian service. Born to Irish immigrants in Australia, Cashin trained at St. Vincent's Hospital, Sydney, before expanding her expertise in London and joining the British Red Cross during World War I. Her service included critical roles in France and Egypt, and she was eventually promoted to 'seas-matron' on the HMHS Gloucester Castle. During a torpedo attack by a German U-Boat, Cashin displayed extraordinary bravery, overseeing the evacuation of over 399 patients and ensuring their safety before leaving on the last lifeboat. After the war, she managed a military hospital in England before returning to Australia to care for her ailing father and later her elderly uncle. Cashin's post-war years included a modest stint running a stationery shop and her retirement in Victoria Road. Her exemplary service earned her multiple accolades, including the Star Medal and the Royal Red Cross Medal, the latter being the first awarded to an Australian. She was also honoured with multiple mentions in dispatches and personal invitations to Buckingham Palace. Alice Cashin's legacy is memorialised at the Woronora Cemetery, with her medals and accolades displayed at the ANZAC Memorial in Sydney, reflecting her enduring impact on the nursing profession and her remarkable dedication to service and care.

Standing tall, this statue of Alice Cashin at the Woronora Cemetery, her place of burial in New South Wales, Australia aims to serve as an everlasting testament to the dedication she displayed towards the provision of service and care to her patients, both during times of conflict and throughout her selfless personal endeavours (Figure 1).^1^

Alice Alanna Cashin was born on the 26th of March, 1870, to Richard Cashin and Catherine Meehan Cashin. She came from a family of immigrants from Ireland who had migrated to Australia to alleviate labour shortages. She completed her early education at a women's private institution in Sydney. Alice's passion for care and service led her to pursue nursing at St Vincent's Hospital in Darlinghurst, where she underwent training from 1893 to 1896; she stayed on for another year as a certified nurse, following which she ventured into private nursing, where she worked as a ‘nurse for hire’ in Sydney, Richmond and Lismore.^2^ Alice later joined the Australasian Trained Nurses’ Association on the 30th of July, 1901. In 1909, Alice left for London, UK where she pursued a diploma in Therapeutic Massage at the International School of Therapeutic Massage.^3^ Although her intentions were always to return home to Australia, the outbreak of the First World War led to Alice offering her services to the British Government, and embarking on a nursing journey at the General Hospital in Calais, France with the British Red Cross. Here, she tended to the wounds of soldiers, as well as Belgian refugees escaping oppression.^4^ It was also then that she changed her middle name from the Irish ‘Alanna’ to the English/Norman ‘Elanor’; she would revert back to the former after her return to Australia many years later.^3^ On the 19th of July, 1915 she returned to England and joined the Queen Alexandra's Imperial Military Nursing Service Reserve and consequently went on to assume responsibility for a sizable surgical ward in the general hospital located in Ras-el-din, Egypt.^2,3^

**Figure 1.**
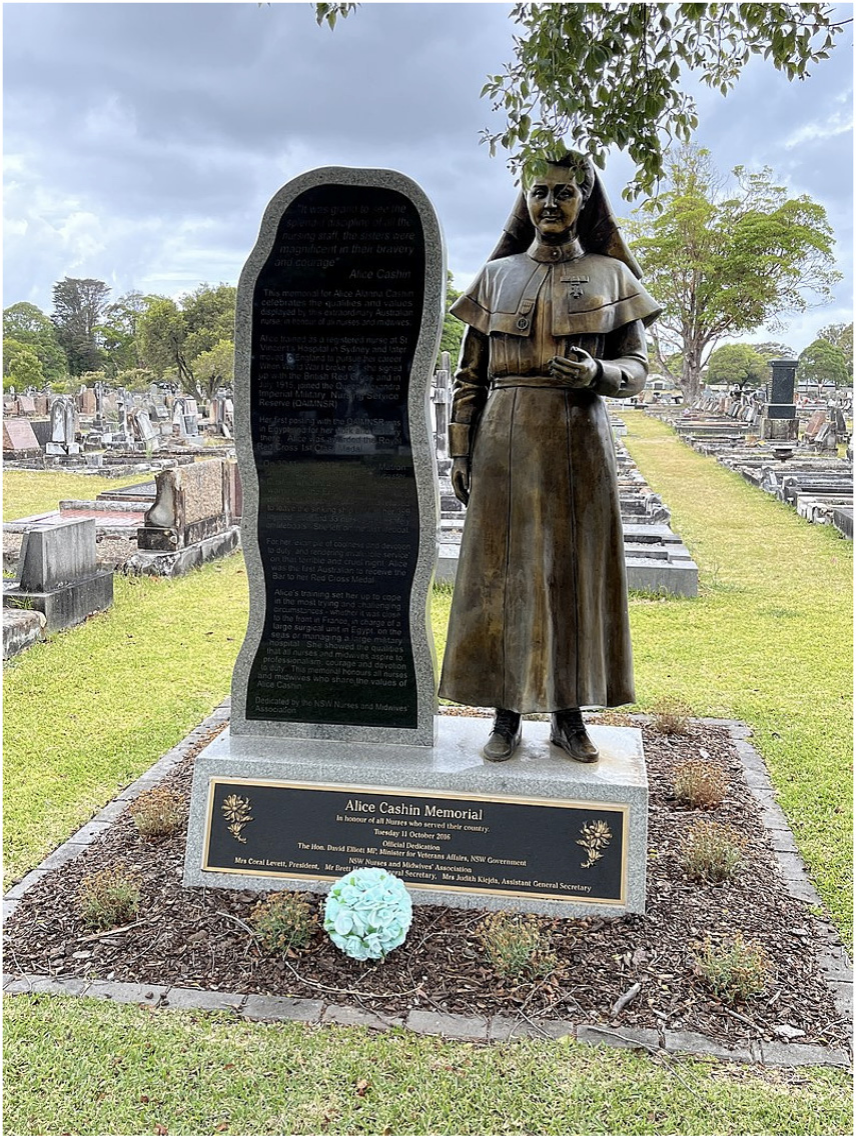
The State of Alice Cashin in Woronora Cemetery in New South Wales, Australia.^1^

In 1916, in recognition of her exemplary efforts in France and Egypt, Alice was promoted to ‘seas-matron’ aboard the HMHS Gloucester Castle (a British steamship requisitioned for use as a Hospital Ship for the British Forces), where she was placed in-charge of a number of nurses onboard the ship. Collectively, the team was responsible for catering to the wounds of severely wounded British soldiers. The HMHS Gloucester Castle travelled between England and a number of ports, including Salonika (Greece), Malta, Cyprus and Egypt amongst many more.^3^ On the night of the 30th–31st of March, disaster struck as the HMHS Gloucester Castle was torpedoed by a German U-Boat (SM-UB32) off the Isle of Wight, without any warning signs. Alice swiftly sprang into action, taking charge and providing guidance during the implementation of emergency protocols. Onboard the sinking ship, Alice, alongside a team of soldiers, diligently worked to safely evacuate more than 399 patients, including 200 bedridden individuals who were unable to move. Collaborating with her fellow nurses, she efficiently distributed pre-prepared bags containing dressings, sedatives, medications, and blankets, ensuring that the necessary supplies were available to provide comfort and care to those being evacuated. In such situations, women were instructed to evacuate early. However, Alice disobeyed all orders as she ensured the rest of those onboard were safely evacuated before she left on the very last lifeboat, with just ‘her crucifix, prayer book and cape gifted to her by Her Majesty Queen Alexandra’ along with a few items.^4–6^ Following the incident, rather than return to sea, she was placed in-charge of a 400-bed military hospital at Whittingham Barracks in Litchfield, England where she remained for two years.^2^

In 1919, Alice made the decision to return to her homeland of Australia owing to her father's deteriorating health. She established her residence at Moore Park, where she devoted herself to caring for her father and his third wife. Unfortunately, her father passed away six months after her arrival. Following his death, Alice continued nursing, albeit on a more personal level. She subsequently found a new home with her elderly uncle, Jeremiah, who resided in Marrickville's Queen Street. Her stay with him lasted until his demise in 1922; she cared for him during his last few years. Following on, she opened a stationery shop (for someone who had been commended by the British Government and the Queen for service; this was quite a humble transition). At the age of 66, in 1933, Alice relinquished ownership of the shop and went to retire at her last home in Victoria Road.^2,3^ She died on the 4th of November 1939 from chronic nephritis and was buried at the Woronora Cemetery. She had never married.^2^

Throughout her illustrious nursing career, Matron Alice Cashin received a multitude of accolades. Notably, she earned mention in dispatches on two occasions (which is when a superior officer drafts an official report to the high command in recognition of an individual's gallantry).^2,3,7^ In 1914, she was awarded the Star Medal, and subsequently went on to be awarded the Royal Red Cross Medal in 1917, and was the first Australian citizen to do so. Her dedication to duty and exemplary service also granted her the privilege of being invited for tea with the King and Queen at Buckingham Palace on four separate occasions.^2,3^ Furthermore, Alice's contributions were acknowledged by Sir Walter Davidson, the then Governor of New South Wales.^3^ Presently, her esteemed medal and accolades are proudly exhibited for public viewing at the ANZAC Memorial in Sydney, and continue to serve as an inspiration to many aspiring nurses.^8–10^
